# Insights on Transmission, Spread, and Possible Endemization of Selected Arboviruses in Israel—Interim Results from Five-Year Surveillance

**DOI:** 10.3390/vetsci9020065

**Published:** 2022-02-02

**Authors:** Adi Behar, Orly Friedgut, Ditza Rotenberg, Olga Zalesky, Omer Izhaki, Amit Yulzary, Asael Rot, Ricardo Wolkomirsky, Lior Zamir, Faris Hmd, Jacob Brenner

**Affiliations:** 1 Division of Parasitology, Kimron Veterinary Institute, Bet Dagan 50250, Israel; orlyf@moag.gov.il (O.F.); ditzar@moag.gov.il (D.R.); olgaz@moag.gov.il (O.Z.); omerit1212@gmail.com (O.I.); amit.yulzary@mail.huji.ac.il (A.Y.); rotasa@gmail.com (A.R.); brennerjacovet@gmail.com (J.B.); 2 Veterinary Field Services, Bet Dagan 50250, Israel; ricardow@moag.gov.il (R.W.); liorz@moag.gov.il (L.Z.); faresh@moag.gov.il (F.H.)

**Keywords:** *Culicoides*, *C. imicola*, *C. oxystoma*, *C. puncticollis*, Simbu serogroup viruses, bovine ephemeral fever virus, bluetongue viruses, epizootic hemorrhagic disease viruses, monitoring, surveillance and early warning systems

## Abstract

Outbreaks of arthropod-borne (arbo) viruses that infect livestock impact the health and welfare of domestic and wild animals are often responsible for significant economic losses in livestock production. Surveillance and early warning systems effectively predict the emergence and re-emergence of arboviral disease. This paper presents the interim results of five years monitoring the exposure of sentinel naïve heifers and *Culicoides* biting midges (Diptera; Ceratopogonidae) to bovine ephemeral fever virus (BEFV), Simbu serogroup viruses, bluetongue viruses (BTV), and epizootic hemorrhagic disease viruses (EHDV). The data were collected from 11 dairy farms situated within eight different geographical regions in Israel. The results indicate that cattle in Israel are affected by all four viruses from the early summer onward. The investigated viruses exhibit unique site-specific profiles in both ruminants and vectors. The potential of several vectors to transmit these viruses and lack of cross-protection to re-infection with multiple serotypes (BTV and EHDV) or species (Simbu serogroup viruses) highlights some likely mechanisms that may play a role in these viruses’ maintenance cycle and possible endemization in our region.

## 1. Introduction

Outbreaks of arthropod-borne (arbo) viruses that infect livestock impact the health and welfare of domestic and wild animals are often responsible for significant economic losses in livestock production. Unveiling the patterns of viral spread is difficult, as their epidemiology is the result of the integrated interactions between several factors: the virus, vertebrate host, vectors, and environment. The ability of a virus to infect, replicate in, and be transmitted by the vector(s) into a new host, the genetics of both pathogen(s) and vector(s), the virulence of the pathogen(s), the immune status and genetics of the host(s) are all crucial in determining the course of the infection and its results. Global trends such as the changing climate and ecological conditions, trade, and human behavior are triggering the emergence and re-emergence of arboviral diseases. Surveillance and early warning systems effectively predict the emergence and re-emergence of vector-borne disease. By targeting vector populations and sentinels, these systems can successfully identify areas where pathogens are circulating, provide accurate data for epidemiological models, assist in predicting the course and timing of infectious disease, and simulate the impact of control strategies such as vaccines, for example [1].

In 2015, an arboviruses monitoring system was established at the Kimron Veterinary Institute (KVI). Monitoring includes serum sampling of naïve sentinel heifers and trapping of *Culicoides* biting midges (Diptera; Ceratopogonidae). The monitoring system objectives are: (a) To obtain insight into the spread and incidence of selected arboviral diseases and (b) to supply an early warning to farmers and policymakers. Representatives of four arbovirus families, the *Rhabdoviridae* (bovine ephemeral fever virus (BEFV)), the *Peribunyaviridae* (Simbu serogroup viruses), and the *Reoviridae* (bluetongue (BTV) and epizootic hemorrhagic disease (EHDV) viruses) were selected for monitoring.

Bovine ephemeral fever virus (BEFV) is a noncontagious, negative-sense single-strand (ss) RNA. Its genome encodes five structural proteins (N, a nucleoprotein; P, a polymerase-associate protein; M, a matrix protein; L, a viral RNA polymerase-associate protein; and G, a surface glycoprotein—together with G_NS_, a non-structural glycoprotein). The virus is suspected to be transmitted by insects, and it has been isolated from a variety of insect vectors, including *Culicoides* biting midges and mosquitoes [2,3]. This virus’s most characteristic clinical manifestation in cattle is an eruption of high body temperature in many animals simultaneously; characteristically, clinical signs last three days. The disease is therefore known as a three-day sickness. BEF can cause heavy economic losses, mainly reflected by a sharp milk yield drop. If BEF is not treated promptly, returning to the milk yield before the infection is cumbersome and sometimes remains lower than expected during the ongoing milking [2]. Moreover, other disease outcomes might be a reduction in herd prolificacy, lower fertility in bulls, and fatality. Until 2015, four significant outbreaks occurred in Israel, the first starting in 1999 among dairy-cattle herds in the Jordan Valley, from where it spread to the Mediterranean Coastal Plain [4]. The second outbreak started in 2004 and was much more widespread, covering most of Israel’s Mediterranean Coastal Plain [4,5]; the third, in 2010, covered the Interior Plain [4]; and the last one was recorded in 2014–15 [5]. Commercial attenuated and killed vaccines are available to farmers.

Simbu serogroup viruses are among the largest serogroups within the genus *Orthobunyavirus* of the family *Peribunyaviridae*. They comprise at least 25 antigenically different but serologically related negative sense (−) single-stranded (ss) RNA viruses transmitted mainly by *Culicoides* biting midges. Some of them are responsible for outbreaks of congenitally deformed ruminant neonates, the arthrogryposis-hydranencephaly syndrome (AH-S) [6]. The group of ruminants at risk are pregnant dams. Abortions, dystocia, neurological, and rabies-like manifestations might be observed in infected adult ruminants [6,7,8]. Several viruses from this serogroup have been shown to cross the placenta of ruminants to the developing fetus—Akabane (AKA), Satuperi (SAT), Aino (AINO), Shamonda (SHA), Shuni (SHU), Peaton (PEA), and Schmallenberg (SB) viruses. Replication of these viruses in the developing fetus can cause outbreaks of abortion, stillbirth, and malformations that are only seen at birth [6,7]. The neonatal skeletal malformations are known as arthrogryposis, and the neurological damages can range from microscopic to mild or severe. It may include hydranencephaly, microencephaly, and polio-encephalomyelitis. The damages are correlated with the stage of pregnancy at which the mother is infected. For instance, severe brain malformations in cattle may occur if the naïve female is infected between 76 and 106 days of pregnancy [6,7]. In Israel, AH-S outbreaks related solely to AKAV infections were documented in 1969–1970, 2001–2002, and 2012. Additional AH-S vast outbreaks in 2014/15 were connected with SHUV infections. Further studies revealed the presence of other Simbu viruses: SAT, SHA, PEA, SB, and Sango (for a recent review, see [8]). Vaccines against Simbu serogroup viruses are not available to Israeli farmers.

BTV and EHDV are the two orbiviruses implicated in ruminant diseases in Israel. These are segmented double-stranded RNA viruses causing varying hemorrhagic diseases ranging from subclinical to severe in farm ruminants. Viraemia can be prolonged, despite neutralizing antibodies, due to an intimate association between viruses and erythrocytes, thus facilitating horizontal transmission by *Culicoides* [9]. Monitoring BTV and EHDV in sentinels is warranted by the OIE.

Based on sero-neutralizing activity, more than 30 different BTV serotypes are recognised [9]. BTV infect all domesticated and wild ruminant species; however, severe clinical disease or death from BTV infection is mainly restricted to sheep. Although BT disease has an 80-year history in Israel, it is primarily reported in sheep [10]. Multiple serotypes of BTV have been identified in Israel. Up to 2000, five serotypes (BTV-2, 4, 6, 10, and 16) had been confirmed [10]. From 2008, more serotypes have been identified. In some cases, up to three distinct BTV serotypes were found co-circulating on the same farm and even in the same animal [11,12]. The serotypes that have been added to the Israeli virus repertoire were BTV-3, 5, 8, 9, 12 15, 16 (the eastern type that “replaced” the western type), and 24 [13,14]. BTV-24 was isolated during an epidemiological investigation of an extensive nervous–like syndrome (shaky lambs) outbreak in southern Israel [11]. BTV-8 caused an outbreak in northern Israel and was isolated for the first time at the end of 2008. In 2010, it spread throughout Israel and caused outbreaks among sheep, cattle, and goats [11]. BTV-3 was isolated for the first time in 2013. Until 2018, it was limited to sheep flocks in the Negev Desert (Southern Israel); however, during 2018, it spread throughout Israel, affecting all ruminants [13].

Lastly, an unidentified BTV serotype, isolated from an imported commercial vaccine batch used to immunize cattle against lumpy skin disease in 2017, was later identified as BTV-28 [15]. Commercial vaccines are available only for BTV-4 and BTV-8.

EHDV is closely related to BTV. There are seven known serotypes of EHDV to date. Some serotypes are more virulent to cattle than others, mainly the Ibaraki virus, which causes sporadic disease outbreaks in cattle in Asia and is now considered EHDV-2 (serotype 2) [9]. However, it seems that the epidemiology of EHD is shifting in recent years as outbreaks among cattle have been reported with elevated frequencies in the Mediterranean Basin, South Africa, Reunion Island, and the USA, mainly due to infections with EHDV-2, 6, and 7 [9]. In Israel, three major outbreaks were documented: EHDV-7 in 2006 [16], EHDV-6 in 2015 [17], and EHDV-1 in 2016–2017 [18]. Both EHDV-6 and EHDV-7 outbreaks started in the Jordan Valley and later spread countrywide [16,17]. Vaccines against EHDV are not available to Israeli farmers.

This paper presents the interim results of five years monitoring the exposure of sentinel naïve heifers and *Culicoides* biting midges to BEF, Simbu serogroup viruses, BTV and EHDV. The data were collected from 11 dairy farms situated within eight different geographical regions in Israel. Accordingly, we describe the spread and incidence of these viruses’ infections in sentinel naïve dairy cattle and infected *Culicoides* and discuss the possible endemization of emerging and/or newly introduced arboviruses in our region.

## 2. Material and Methods

### 2.1. Study Area

**Figure 1:**
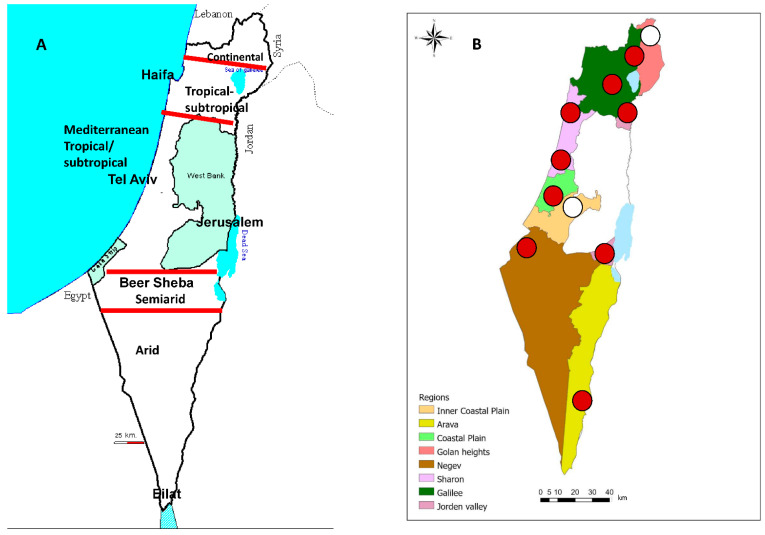
Map of Israel. (**A**). Israeli different climate zones, (**B**). Locations and types of farms sampled in this study. In white: farms that were thought to be “Negative controls” because they reported no clinical signs attributed to arboviral diseases until 2015. In red: all other farms.

The Israeli monitoring system consists of 11 dairy farms situated within eight geographical regions of Israel: the Arava (29.5–30.5° N) (1 farm), the Negev Desert (29.7–30.7° N) (1 farm), the South Jordan Valley (31.5° N) (1 farm), the Interior Plain (31.9° N) (1 farm), the Coastal Plain (31.9° N) (1 farm), the Sharon Plain (32.2° N) (2 farms), Galilee, including the North Jordan Valley (32.7–33.5° N) (3 farms), and the Golan Heights (34.1° N) (1 farm) (Figure 1A,B). The Negev and Arava and the South Jordan Valley are arid zones that receive very little rain and extreme temperature due to their location east of the Sahara. In contrast, other regions enjoy a Mediterranean climate (i.e., hot, dry summers (average Tm of 26 °C) and cool, mild winters (average Tm of 14 °C)) (Figure 1A). However, in being mountainous, the Golan Heights are generally cooler and wetter. The highest dairy farm in Israel, located at the Golan Heights, is 950 m above sea level. The second highest dairy farm is located on the Judea Mountains at 800 m above sea level. These farms were chosen as “Negative controls” because they reported no clinical signs attributed to arboviral diseases until 2015 (Figure 1B).

### 2.2. Serum Collection

**Figure 2:**
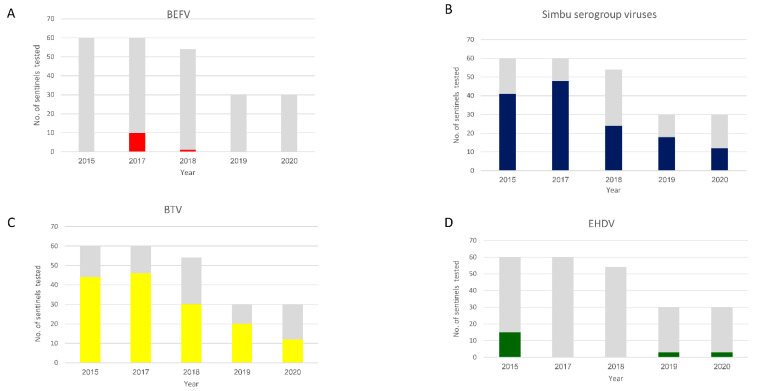
Seroprevalence of the arboviruses studied in sentinel cattle. BEFV (**A**), Simbu serogroup viruses (**B**), BTV (**C**), EHDV (**D**). The grey bars indicate the total of sentinels tested. The colored bars represent seropositive sentinels.

Serum samples were collected monthly between June and December between 2015 and 2020 except for 2016 (Figure 2). At each selected farm, six heifers aged between 6 to 8 months were chosen each year to serve as sentinels throughout the sampling season. All heifers were serologically naïve at the beginning (time 0) of the sampling season and were followed till the end of the sampling season. Upon collection, samples were placed in 4 °C cooling boxes and transported to the laboratory, where they were kept at −20 °C until used for serology.

### 2.3. Insect Collection 

*Culicoides* biting midges were collected using suction light traps equipped with an 8 W black light and a downdraft suction motor powered by two rechargeable 1.5 V GP2700 AA batteries. Insects were collected into a reusable plastic jar suspended below the trap’s fan. On the afternoon before serum sampling, two light traps were placed overnight (1 h before sunset and retrieved one h after dawn) at suitable locations on each farm and suspended at the height of 1.7–2 m above the ground. Immediately after collection, the plastic jars containing live insects were placed in 4 °C cooling boxes together with the serum samples and transported to the laboratory. Upon arrival, the live insects were anaesthetized with CO_2_ and sorted into species under a stereoscopic microscope (Nikon SMZ25) using various taxonomic keys [19]. After sorting, live females were grouped in pools (N = 25–50 individuals) according to location and species. None of the individual midges in the pools had any observable blood in their gut. The collected midges were stored at −80 °C until tested for the presence of arboviruses. 

### 2.4. ELISA Serum-Reactivity and Virus Neutralisation Test (VNT)

- **BEFV**—Cell lines containing the local virus strain (the complete genome sequence was published in [20]) were used for VNT [21].

For the other viruses, commercial ELISA kits were used according to the manufacturer’s instructions:

ii. **Simbu serogroup viruses**—IDEXX Schmallenberg Ab’s Test Kit (3097 Liebefeld-Bern, Switzerland) was used.
iii. **BTV** and **EHDV**—IDVET—ID Screen^®^ bluetongue and IDVET—ID Screen^®^ EHDV Competition (Montpellier, France) were used, respectively.

### 2.5. Genomic Detection of Arboviruses in Culicoides

- **Viral RNA extraction and cDNA synthesis from *Culicoides:***

Pooled *Culicoides* were homogenized in 500 µL sterile phosphate-buffered saline (PBS) using a rotor-stator homogenizer for RNA extraction. A 200 µL aliquot of the homogenate was used for total viral nucleic acid extraction with the Maxwell 16 Viral Total Nucleic Acid Purification Kit (Promega, Madison, USA) according to the manufacturer’s instructions. The remaining 300 µL was kept at −80 °C for later use. According to the manufacturer’s instructions, total viral nucleic acids (0.4 µg) were used for cDNA synthesis using UltraScript Reverse Transcriptase (PCR Biosystems Ltd., London, UK).

ii. **RT-nested PCR** and **RT-nested qPCR amplifications:**

During our molecular work with all the targeted viruses, our internal quality controls indicated that while performing regular PCRs and qPCRs using previously published primers, we got a higher frequency of false positives because of a non-specific amplification of *Culicoides* ribosome (18 s rRNA) from the *Culicoides* homogenates. Moreover, we discovered that *Culicoides* homogenates have a high inhibitory effect. Consequently, we added a second amplification step to increase the specificity of our detection systems as follow:

**ii (a) BEFV:**

cDNA synthesis and subsequent initial RT-qPCR amplification targeting the BEFV Protein G gene segment were carried out, according to Erster et al. (2017) [5]. To provide higher specificity, a nested qPCR was designed as follows: initial rounds of amplification were performed in 25 μL reactions containing 4 µL template cDNA and 1 μM external forward and reverse primers (807F-5′-CCAGGTTTCAGAATGCACAC-3′ and 1500R-5′-CTCTCACTATATCAGTTCTG-3′) in a conventional thermocycler for 10 cycles of amplification. For the second round of amplification, 2 μL of the PCR product from the initial amplification was used as the template in a qPCR containing 0.1 µM internal primers, according to Erster et al. [5]. Known concentrations of BEFV were used as the external standard. A negative control (no reverse transcriptase added) and positive control (BEFV) were run in parallel. All samples were run in duplicate. Reactions were performed on a CFX96 Touch Real-Time PCR Detection System (Bio-Rad, Hercules, CA, USA) with the manufacturer-recommended PCR parameters. Samples with a cycle threshold (Ct) value < 30 and melting temperature (Tm) of 76 °C–80 °C were suspected of being BEFV positive [5].

**ii (b) Simbu serogroup viruses:**

cDNA synthesis and subsequent nested qPCR and nested PCR were conducted according to Behar et al. [19].

**ii (c) BTV-8 and BTV-4:**

This study focused only on detecting BTV serotypes to which commercial vaccines are available, i.e., BTV-4 and BTV-8. cDNA synthesis and subsequent initial PCR amplification with specific primers targeting the serotype-specific genome Segment 2 (seg—2) of BTV-4 and BTV-8 was conducted according to Maan et al. [22]. To provide higher specificity, RT-nested PCRs were performed using primers previously published by Maan et al. [22] as follows: 

For BTV-4 amplification, RT-PCR targeting BTV-4 seg-2 was conducted using external primers BTV-4/2/106F and BTV-4/2/919R. The PCR products served as the template for the nested PCR with primers BTV-4/2/818-837F and BTV-4/2/841R (see Table S1 in [22]) (Expected product size ca. 1706 bp). 

For BTV-8 amplification, RT-PCR targeting BTV-8 seg-2 was conducted using external primers BTV-8/2/101F and BTV-8/2/870R. The PCR products served as the template for the nested PCR with primers BTV-8/2/276F and BTV-8/2/500R (see Table S1 in [22]) (expected product size ca. 706 bp). 

No attempts were made to detect EHDV from *Culicoides*.

In all the amplifications stated above, negative controls (no DNA added) were always performed in parallel. Positive controls were only added to the final nested step to avoid contamination. All samples were amplified in a conventional PCR (SensoQuest Labcycler, Goettingen, Germany) with 1 µM primer and addition of 10 ng/µL bovine serum albumin (BSA) in 2X PCRBIO HS Taq mix (PCR Biosystems Ltd.) for a total volume of 25 µL reaction mixture according to the manufacturer’s instructions. Bovine serum albumin was added to improve PCR amplification as the amounts of viral nucleic acid extracted from *Culicoides* and ruminant samples were relatively low. Products were separated on a 1.5% (*w*/*v*) agarose gel in TAE buffer (40 mM Tris-acetate, 1 mM EDTA) and stained with SmartGlow PS (Accuris Instruments, Edison, NJ, USA). All PCR products were sequenced in both directions. Sequences chromatograms were visually inspected, verified, aligned, and annotated using Geneious Pro (Biomatters, NJ, USA) (https://www.geneious.com, available on 26–27 December 2021).

Levels of intraspecific sequence homology of the 16 sequences of BTV-8 and the 2 sequences of BTV-4 were consistently high (99.0–99.5%); accordingly, only a single reference sequence from each host was selected and deposited in GenBank under accession numbers OM033723-OM033726.

## 3. Results

### 3.1. Detection of Antibodies against Bovine Arboviruses

All the arboviruses investigated in this study were serologically detected (Figure 2). In total, 234 sentinels were monitored during the study: 60 in 2015 and 2017, 54 in 2018, and 30 in 2019 and 2020.

**Figure 3:**
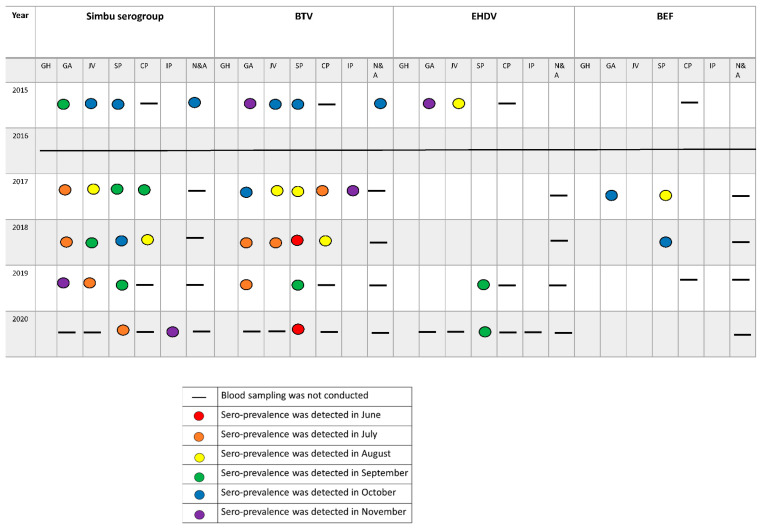
Seroprevalence of arboviruses in sentinel cattle by each geographical area between 2015 and 2020. GH = Golan Heights, GA = Galilee (including North Jordan Valley), JV = South Jordan Valley, SP = Sharon Plain, CP = Coastal Plain, IP = Inner coastal plain and the Judea Mountains, N&A = Negev and Arava deserts.

- **BEF**—Neutralizing antibodies against BEFV were detected in 2017 in 10 of 60 animals (Figure 2A) from 2 farms: one from Galilee and the other from the Sharon Plain. Only one sentinel from a farm in the Sharon Plain was VNT positive in 2018 (Figure 2A and Figure 3). These 11 sentinels seroconverted during summer or early autumn (Figure 3).
- **Simbu serogroup viruses**—In total, 143 out of 234 sentinels seroconverted against Simbu serogroup viruses during this study. Forty-one in 2015; 48 in 2017; 24 in 2018; 18 in 2019 and 12 in 2020 (Figure 2B and Figure 3). Seroconversion mostly accrued during the hot months (Figure 3). Seroconversion occurred in all the farms examined except for the farms located at the Golan Heights and the Judea Mountains during 2015–2019. In 2020, seroconversion occurred in all the farms examined, including a single animal from the farm in the Judea Mountains. The farm in the Golan heights remained “naïve” throughout the study (Figure 3).
- **BTV**—In total, 152 out of 234 sentinels seroconverted against BTV during this study. Forty-four in 2015; 46 in 2017; 30 in 2018; 20 in 2019 and 12 in 2020 (Figure 2C and Figure 3). Seroconversion mostly accrued during the hot months (Figure 3). Seroconversion occurred in all the farms examined except for the farms located at the Golan Heights and the Judea Mountains during 2015, 2018–2020. In 2017, seroconversion occurred in all the farms examined, including a single animal from the farm in the Judea Mountains. The farm in the Golan Heights remained “naïve” throughout the study (Figure 3).
- **EHDV**—In total, 21 of 234 sentinels seroconverted against EHDV during this study. During 2015, 15 of 60 sentinels became EHDV seropositive (Figure 2D) from 3 farms: A farm from the northern Jordan Valley (N = 5), a farm from the Southern Jordan Valley (N = 5), and a farm from around the Sea of Galilee (N = 5) (Figure 3). In 2019 and 2020, EHDV was detected in 3 of 30 sentinels each year (Figure 2D) from a farm in the Sharon Plain (Figure 3). EHDV seroconversion accrued mainly during the summer (Figure 3).

### 3.2. Genomic Detection of BEFV, Simbu Serogroup Viruses, BTV-4 and BTV-8 in Culicoides

In total, 118 pools (each pool contained 25–50 midges) were analyzed: 48 of *C. imicola* Kieffer, 1913 (subgenus *Avaritia* Fox), 32 of *C. oxystoma* *C. oxystoma* Kieffer, 1910 (subgenus *Remmia*), 26 pools *C. puncticulis* Becker, 1903 (subgenus *Monoculicoides* Khalaf) and 12 of *C. newsteadi* Austen, 1921 (subgenus *Culicoides* Latreille). These species were selected as they were shown to be the dominant *Culicoides* spp. around livestock farms in Israel [19].

- **BEFV** was not detected in any of the 118 pools tested in this study.
- **BTV-4** was detected in only two pools of *C. imicola*, one collected during 2015 and one during 2016 (Table 1). Both pools originated from the same farm in the Northern Jordan Valley during autumn.
- **BTV-8** was detected in 16 pools from three out of the four *Culicoides* species tested (i.e., *C. imicola*, *C. oxystoma*, and *C. puncticulis*). We found BTV-8 positive pools in all the geographical regions, except the Golan Heights, only during 2015–2017 (Table 1).
- **Simbu serogroup viruses** detection in *Culicoides* between 2015–2019 was previously published [19,23,24]. Thus, only pools collected during 2020 were analyzed for this study. Simbu serogroup viruses were detected in 12 pools collected during 2020. Four pools collected from the Sharon Plain were positive to AINOV, and the other eight were positive to AKAV (Table 2).

## 4. Discussion

The Israeli arboviruses monitoring system established in 2015 has two objectives:

The first objective is to obtain insight into the spread and incidence of arboviruses affecting ruminants. The second objective is to supply an early warning to farmers and policymakers so that they can be adequately prepared for the arrival of such diseases through vaccinations (if available) or changes in their livestock farming practices (for example, slight shifts in breeding times to months with a lower risk of infection; changing grazing interfaces and replacing plots, etc.). Therefore, we focused our efforts either on arboviruses recommended by the OIE (i.e., BTV and EHDV) or on arboviruses that the incidence of the diseases caused by them increased in the last 20 years (i.e., BEF and Simbu serogroup viruses).

- **Livestock in Israel are exposed to all investigated viruses from early summer onward.**

Our five-year interim surveillance results provide new insight into the spread and incidence of the selected arboviruses in sentinel naïve dairy cattle and *Culicoides* populations. Our results from sentinel cattle indicate that in Israel, livestock is exposed to all investigated viruses from early summer onward (Figure 3). The livestock experienced BTV and Simbu serogroup viruses’ infections annually whereas, EHDV and BEF outbreaks occurred more sporadically (Figure 2). We were unable to detect BEF in vectors, and consequently, we could not complete and corroborate BEF and EHDV outbreaks with molecular data regarding their presence in vectors. Nevertheless, during this monitoring period, BEFV was diagnosed in cattle only during 2017–2018 (KVI’s annual reports), and two outbreaks of EHDV were reported: EHDV-6 outbreak in 2015 [17] and EHDV-1 in 2016–2017 [18]. The EHDV-6 outbreak was confirmed in late September 2015 in dairy farms from two different geographic locations along the Jordan Valley and, later on, was reported to spread countrywide [17]. The 15 sentinels found to be seropositive to EHDV in our study were from the exact two geographic locations in which the outbreak was initially recognized ([17], Figure 2). The EHDV-1 outbreak was clinically observed from mid-September 2016–Feb 2017 countrywide [18]. Unfortunately, during 2016, serum samples were not collected due to technical issues. The sentinel heifers chosen for 2017 monitoring were born in late January 2017 and were either not exposed to the virus or had maternal antibodies protecting them. Therefore, none of the sentinels from 2017 were shown to be positive to EHDV.

ii. - **Each farm should be considered its own microhabitat as all investigated viruses exhibit unique site-specific profiles in both ruminants and *Culicoides* vectors.**

Notably, it appears that all four investigated viruses exhibit unique site-specific seroreactivity profiles in ruminants and display more of a “quilt” or “patches” rather than a carpet pattern (Figure 3). A similar pattern was previously described for Simbu serogroup viruses in a serosurvey conducted in Israel one year before the beginning of the monitoring [25] and in South Africa for the *Culicoides* transmitted equine encephalosis virus (EEV) and African horse sickness virus (AHSV) [26,27,28]. This “quilt” pattern is also observed within *Culicoides* vectors. Our results show that several *Culicoides* species (i.e., *C. imicola*, *C. oxystoma*, and *C. puncticulis*), each with its own ecology, present the potential for vectoring BTV-8 in the field in various combinations depending on the geographical area from which they are collected (Table 1). Additionally, our results show that BTV-4 and BTV-8 were circulating in the same farm simultaneously (Table 1), corresponding with previous observations of infection of Israeli ruminants with different BTV serotypes in the same farm even in the same animal [11,12]. Previously published data collected during this monitoring period on *Culicoides* populations and the detection of Simbu serogroup viruses in them between 2015–2019 revealed similar results [19,24]. Therefore, we hypothesize that the “quilt” pattern observed in the viral cycling between different competent vectors and susceptible sentinels indicates that each farm should be considered its own “ecological pocket” or microhabitat.

iii. **Possible mechanisms of disease ‘endemization’ and local emergence of arboviral diseases in Israel.**

At the beginning of this study, two such “pockets” were recognized: one farm located at the Golan Heights and the other situated on the Judea Mountains. These farms were thought to be “Negative controls” because they reported no clinical signs attributed to arboviral diseases until 2015. While clinical cases of all four arboviruses monitored in this study were reported from different farms located in the central and south parts of the Golan Heights ([18,24], KVI’s annual reports), the farm on the Golan Heights remained a naïve pocket. Our results also showed that sentinel animals from the farm located on the Judea Mountains were exposed to BTV during 2017 and to Simbu serogroup viruses in 2020. These serological exposures were corroborated with sporadic, laboratory-confirmed clinical cases from the farm. Therefore, we suspect that while the farm from the Golan Heights was naïve up till 2020, it is only a matter of time until arboviral diseases will also be detected in this area. Indeed, there is a dire need to understand better the possible mechanisms of disease ‘endemization’ and affected site re-emergence of arboviral diseases. It appears that local emergence is usually driven by a combination of environmental variables that are not yet completely understood but affect vectors and hosts abundance through space and time [29]. In each farm or a microhabitat presented here, variables such as climate change, inadequate infrastructures, inadequate waste and water management, animal overpopulation, and unregulated animal trade and transportation from one farm to another may influence the prevalence of all arboviruses investigated during specific outbreaks or within specific geographical areas.

Nevertheless, we postulate that once a virus or a new serotype enters and emerges in a particular farm, it is there to stay in a cyclic pattern in which the virus might disappear for a certain period, only to reappear at the same infected site a few years later. Recent results from six years of monitoring arboviruses in Poland yielded similar conclusions [30]. Moreover, Paweska and Venter 2004 [28] suggested a parallel theory regarding the mechanisms that are likely to play a role in EEV (another *Culicoides* transmitted segmented virus) natural maintenance cycle and its highly efficient level of countrywide transmission amongst South African horses.

iv. **Israel is an idyllic environment for ‘reassortment’ of distinct serotypes of segmented viruses.**

These microhabitats also provide an idyllic environment for distinct serotypes of segmented viruses (such as EHDV, BTV, and Simbu serogroup viruses) to exchange genome segments either within the vertebrate (ruminant) or invertebrate hosts (*Culicoides* midge) by a process of ‘reassortment’ allowing these viruses to rapidly evolve and adapt to local conditions and ecosystems. Combined with a lack in cross-protection to re-infection with multiple serotypes (BTV and EHDV) or species (Simbu serogroup viruses), more dangerous biological properties of these viruses, including persistence, virulence, and distribution, are likely to occur and emerge within both vector and host populations in the Israeli farms.

Finally, the second objective of our monitoring was to supply an early warning to farmers and policymakers. In 2018, our surveillance system achieved this objective. It detected the Schmallenberg virus (SBV) entrance to Israel and warned the Israeli National Veterinary Services [24] and the industrialized ruminant farms.

## 5. Conclusions

All investigated arboviruses (i.e., BEFV, EHDV, BTV and Simbu serogroup viruses) affect the Israeli cattle. Each farm is a microhabitat, and once a virus or a new serotype enters and emerges in a particular farm, it stays there in a cyclic arbo-disease pattern. These microhabitats also provide an idyllic environment for distinct serotypes of segmented viruses such as EHDV, BTV and Simbu serogroup viruses to exchange genome segments and create opportunities for reassortment, thus allowing these viruses to rapidly evolve and adapt to local conditions.

## Figures and Tables

**Table 1:** BTV-4 and BTV-8-positive vector pools collected during monitoring in dairy farms between 2015–2020.

Geographic Region	Date of Collection	Vector Species Tested	BTV-4/BTV-8 Detection
Northern Jordan Valley	Sep-2015	*C. puncticulis*	-/+
	Oct-2015	*C. puncticulis*	-/+
	Nov-2015	*C. imicola*	+/+
	Oct-2016	*C. imicola*	+/+
Interior Plain	Jul-2015 (x2)	*C. imicola* (x2)	-/+
	Jul-2015	*C. oxystoma*	-/+
	Sep-2017	*C. oxystoma*	-/+
Sharon	Aug-2017	*C. imicola*	-/+
	Aug-2017	*C. oxystoma*	-/+
	Aug-2017	*C. puncticolis*	-/+
Coastal Plain	Nov-2015	*C. imicola*	-/+
	Nov-2015	*C. oxystoma*	-/+
Negev	Jul-2017	*C. puncticolis*	-/+
Southern Jordan Valley	Aug-2016	*C. oxystoma*	-/+
	Nov-2017	*C. puncticolis*	-/+

**Table 2:** Simbu serogroup viruses-positive pools collected during 2020.

Geographic Region	Month of Insect Collection	Vector Species Tested	Virus Genome Detected
Interior Plain	Oct	*C. imicola*	AKAV
		*C. oxystoma*	AKAV
	Nov	*C. imicola*	AKAV
		*C. oxystoma*	AKAV
Sharon	July	*C. imicola*	AINOV
		*C. imicola*	AINOV
		*C. oxystoma*	AINOV
		*C. puncticolis*	AINOV
	Aug	*C. imicola*	AKAV
Coastal Plain	Sep	*C. imicola*	AKAV
		*C. oxystoma*	AKAV
Negev	July	*C. oxystoma*	AKAV

## Data Availability

Essential data supporting the conclusions of this article are included in the main text.
